# Genetic Association and Differential RNA Expression of Histone (De)Acetylation-Related Genes in Pemphigus Foliaceus—A Possible Epigenetic Effect in the Autoimmune Response

**DOI:** 10.3390/life14010060

**Published:** 2023-12-29

**Authors:** Maiara Sulzbach Denardin, Valéria Bumiller-Bini Hoch, Amanda Salviano-Silva, Sara Cristina Lobo-Alves, Gabriel Adelman Cipolla, Danielle Malheiros, Danillo G. Augusto, Michael Wittig, Andre Franke, Claudia Pföhler, Margitta Worm, Nina van Beek, Matthias Goebeler, Miklós Sárdy, Saleh Ibrahim, Hauke Busch, Enno Schmidt, Jennifer Elisabeth Hundt, Maria Luiza Petzl-Erler, Angelica Beate Winter Boldt

**Affiliations:** 1 Laboratory of Human Molecular Genetics, Department of Genetics, Federal University of Paraná (UFPR), Curitiba 81531-980, Brazil; maiarasd@yahoo.com.br (M.S.D.); valeriabumiller@gmail.com (V.B.-B.H.); saralobo5@yahoo.com.br (S.C.L.-A.); gabriel.cipolla@ufpr.br (G.A.C.); dani_malheiros@ufpr.br (D.M.); danillo@augusto.bio.br (D.G.A.); perler@ufpr.br (M.L.P.-E.); 2 Postgraduate Program in Genetics, Department of Genetics, Federal University of Paraná (UFPR), Curitiba 81531-980, Brazil; 3 Department of Neurosurgery, University Medical Center Hamburg-Eppendorf, 20251 Hamburg, Germany; 4 Research Institut Pelé Pequeno Príncipe, Curitiba 80250-060, Brazil; 5 Department of Biological Sciences, The University of North Carolina at Charlotte, Charlotte, NC 28223, USA; 6 Institute of Clinical Molecular Biology (IKMB), Christian-Albrechts-University of Kiel, 24105 Kiel, Germany; m.wittig@mucosa.de (M.W.); a.franke@mucosa.de (A.F.); 7 Department of Dermatology, Saarland University Medical Center, 66421 Homburg, Germany; claudia.pfoehler@uks.eu; 8 Division of Allergy and Immunology, Department of Dermatology, Venerology and Allergy, Charité-Universitätsmedizin Berlin, 10117 Berlin, Germany; margitta.worm@charite.de; 9 Department of Dermatology, University of Lübeck, 23562 Lübeck, Germany; nina.vanbeek@uksh.de (N.v.B.); enno.schmidt@uksh.de (E.S.); 10 Department of Dermatology, Venereology and Allergology, University Hospital Würzburg, 97080 Würzburg, Germany; goebeler_m1@ukw.de; 11 Department of Dermatology and Allergy, University Hospital, LMU Munich, 80539 Munich, Germany; titkarsag.bor@med.semmelweis-univ.hu; 12 Department of Dermatology, Venereology and Dermatooncology, Semmelweis University, 1085 Budapest, Hungary; 13 College of Medicine and Health Sciences, Khalifa University, Abu Dhabi 127788, United Arab Emirates; saleh.ibrahim@uksh.de; 14 Lübeck Institute of Experimental Dermatology (LIED), University of Lübeck, 23562 Lübeck, Germany; hauke.busch@uni-luebeck.de (H.B.); jennifer.hundt@uni-luebeck.de (J.E.H.)

**Keywords:** pemphigus foliaceus, *fogo selvagem*, genetic polymorphisms, RNA expression, post-translational histone modifications

## Abstract

Pemphigus foliaceus (PF) is an autoimmune skin blistering disease characterized by antidesmoglein-1 IgG production, with an endemic form (EPF) in Brazil. Genetic and epigenetic factors have been associated with EPF, but its etiology is still not fully understood. To evaluate the genetic association of histone (de)acetylation-related genes with EPF susceptibility, we evaluated 785 polymorphisms from 144 genes, for 227 EPF patients and 194 controls. Carriers of HDAC4_rs4852054*A were more susceptible (OR = 1.79, *p* = 0.0038), whereas those with GSE1_rs13339618*A (OR = 0.57, *p* = 0.0011) and homozygotes for PHF21A_rs4756055*A (OR = 0.39, *p* = 0.0006) were less susceptible to EPF. These variants were not associated with sporadic PF (SPF) in German samples of 75 SPF patients and 150 controls, possibly reflecting differences in SPF and EPF pathophysiology. We further evaluated the expression of histone (de)acetylation-related genes in CD4^+^ T lymphocytes, using RNAseq. In these cells, we found a higher expression of *KAT2B*, *PHF20,* and *ZEB2* and lower expression of *KAT14* and *JAD1* in patients with active EPF without treatment compared to controls from endemic regions. The encoded proteins cause epigenetic modifications related to immune cell differentiation and cell death, possibly affecting the immune response in patients with PF.

## 1. Introduction

Pemphigus foliaceus (PF) is an autoimmune blistering skin disease endemic in Brazil, where it is also known as *fogo selvagem* (“wild fire”) [1]. PF pathophysiology is distinguished by IgG1 and IgG4 pathogenic autoantibodies produced mainly against desmoglein 1 (DSG1), a transmembrane cell adhesion protein found in keratinocyte cell junction (desmosomes) [1,2]. The skin blisters in PF are caused by keratinocyte separation, mostly in the epidermal granular layer, a process known as acantholysis [2]. Studies trying to unveil the breakdown of the immunological tolerance in PF etiology indicate associations with environmental, genetic, and epigenetic factors [3,4,5]. Continued exposure to mosquito bites is among the candidate environmental factors for EPF [3]. Haplotypes of major histocompatibility complex (MHC) class II genes stand out as one of the most important genetic components: *DR1_DQw1* and *DR4_DQw3* haplotypes have been associated with susceptibility to EPF, while *DR7_DQw2* and *DR3_DQw2* have been associated with resistance against EPF [6]. Hence, certain alleles of single nucleotide polymorphisms (SNPs) in immune system genes have been reported to alter PF susceptibility [4]. Long noncoding RNAs, several of which are involved in epigenetic regulation of gene expression, were also associated with PF susceptibility [7,8,9]. Furthermore, allelic variants of genes involved in epigenetic regulation, whose products are histone lysine demethylase and methyltransferases (*KDM4C*, *SETD7*, *MECOM*, and *PRDM16*), have been associated with PF susceptibility [5]. 

Epigenetic modifications can alter chromatin structure, triggering different patterns of gene expression. Chromatin comprises DNA and histone (H1, H2A, H2B, H3, H4, as well as histone variants) and nonhistone proteins. Covalent post-translational modifications (PTMs) on histone tails, such as (de)methylation, (de)acetylation, and (de)phosphorylation, are established and removed by chromatin-modifying enzymes that integrate multiunit complexes [10]. Among those enzymes, the activity of well-characterized histone acetyltransferases (HATs) and histone deacetylases (HDACs) is correlated with gene activation and repression, respectively [11].

Dynamic epigenetic modifications control immune cells’ development, activation, and differentiation, which affect the development of autoimmune diseases [12]. Abnormal histone acetylation (altered H3/H4 acetylation and/or HDAC expression patterns) has been implicated in autoimmune diseases affecting the skin, such as systemic lupus erythematosus [13,14], alopecia areata [15], and pemphigus vulgaris [16]. In this work, we investigated whether allelic variants of genes encoding HATs, HDACs, and members of complexes that interact with these enzymes influence PF susceptibility and whether these genes are differentially expressed in CD4^+^ T cells. Genetic association and differential RNA expression of histone (de)acetylation-related genes in EPF may indicate a possible epigenetic effect in its autoimmune response, especially related to immune cell differentiation and cell death. 

## 2. Materials and Methods

### 2.1. Association Analysis Samples

**Figure 1:**
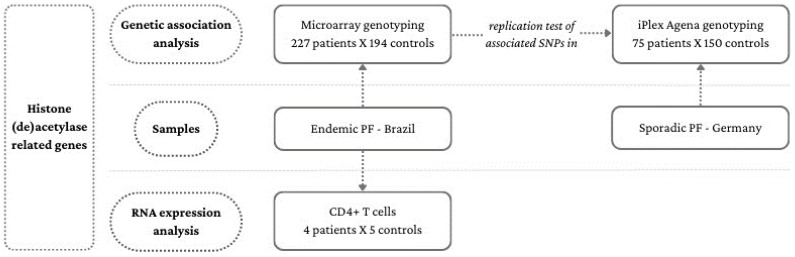
Flowchart of the study samples. The samples, composed of EPF and SPF patients from Brazil and Germany, respectively, and of controls were enrolled for experiments of genetic association with single nucleotide polymorphisms of (de)acetylation-related genes. EPF samples were also enrolled for RNA expression analyses of (de)acetylation-related genes.

Two samples were enrolled in this study—one of EPF and another of sporadic PF (SPF) (Figure 1). DNA from 227 EPF patients and 194 controls was extracted from peripheral blood mononuclear cells (PBMCs) by the phenol–chloroform–isoamyl alcohol method. They lived in Brazilian Midwest, Southeast, and South regions and were recruited in the Hospital Adventista do Pênfigo (Campo Grande, Mato Grosso do Sul), Lar da Caridade—Hospital do Fogo Selvagem (Uberaba, Minas Gerais), Hospital das Clínicas—Universidade de São Paulo (Ribeirão Preto, São Paulo), Hospital de Clínicas—Universidade Federal do Paraná, Hospital de Dermatologia Sanitária São Roque, and Hospital Santa Casa de Misericórdia (Curitiba, Paraná). Patients and controls were unrelated, of predominantly European ancestry [17], and had a similar sex ratio and age distribution: in both groups, 52% were women and the mean age of patients was 40.9 (6–83) years and of controls, 44.8 (11–86) years. No patient had a history of other autoimmune diseases. None of the controls presented autoimmune diseases. 

DNA from 75 SPF patients and 150 controls was isolated from whole blood using the QIAamp DNA Maxi Blood Kit (Qiagen, Hilden, Germany). Patients and controls were all of European ancestry, predominantly of German origin, and recruited in German hospitals by the German Autoimmune Bullous Diseases Genetic Study Group. They had a similar sex ratio and age distribution: 46% of patients and 51% of controls were women and the mean age of patients was 60.1 (25–88) years, and of controls, 60.3 (21–77) years.

### 2.2. RNA Expression Analysis Samples

Total RNA was extracted from CD4^+^ T lymphocytes isolated from peripheral blood samples from four patients (three females and one male), before the start of oral corticosteroid treatment, who constantly presented new skin lesions and whose mean age was 36 (15–54) years, as well as from five controls (four females and one male) from EPF-endemic regions, whose mean age was 39.6 (22–58) years. The selection process of CD4^+^ T cells has been previously described by Bumiller-Bini Hoch et al. [18]. 

### 2.3. SNP Selection and Genotyping

Genotype data were available from a previous study using microarray hybridization (CoreExome-24, version 1.1, Illumina, San Diego, CA, USA) [19]. The genomic positions of 144 genes, encoding HATs (12) and HDACs (18) and members of histone acetylation (59) and deacetylation (55) complexes, were identified, considering 1000 base pairs downstream and upstream from the sequence for the longest transcript, according to the human genome version GRC37/hg19 (NCBI gene) [20] (Table S1). A total of 2486 SNPs located in these gene sequences were extracted from DNA microarray data of EPF samples. 

SNPs associated with EPF were genotyped in the SPF samples using the iPLEX platform of the MassARRAY system (Agena Bioscience, San Diego, CA, USA). The primer sequences are available in Table S2. MassARRAY Typer software (v4.0) (Agena Bioscience, San Diego, CA, USA) with standard settings was used to call the genotypes. The genotype distribution followed the Hardy–Weinberg equilibrium in patients, controls, and the set of participants (data not shown), except for rs13339618 (*p*_controls_ = 0.049; *p*_all participants_ = 0.005).

### 2.4. Association Analysis

Rare variants (minor allele frequency < 0.01) deviating from the Hardy–Weinberg equilibrium for controls (*p* < 0.05) or in high linkage disequilibrium (LD) with another SNP from the dataset (r^2^ ≥ 0.80) were excluded. After filtering, 785 SNPs remained for subsequent logistic regression association analysis with allele frequencies (additive model), frequencies of homozygotes for the minor allele (recessive model), and the sum of the frequencies of heterozygotes and homozygotes for the minor allele (dominant model) (PLINK version 1.1.9), correcting possible associations resulting from population stratification for two principal components of analysis and for sex. *p* < 0.005 was established as significant [21].

### 2.5. In Silico Analysis

Functional annotation available in reference public databases was used to explore the potential effects of the SNPs associated with PF. Gene and SNP annotations were performed with UCSC [22] and Ensembl [23] genome browsers. Gene expression was entrained with Protein Atlas [24], which, in addition to presenting its own database, also exhibits the Monaco dataset [25] and Schmiedel dataset [26]. Allelic variants’ epigenetic annotations were entrained with the HaploReg v.4.1 database [27] and SNPNexus tool [28], which filters many databases, including Roadmap and Encode [29,30]. LD between alleles was evaluated using LDLink [31], with European as a reference. The expression and splicing quantitative trait loci (eQTL and sQTL) effects were observed in Genehopper Qtlizer [32], which compiles information from several databases, such as Blood eQTL Browser [33], GRASP 2 Catalog [34], GTEx v8 [35], The Cardiogenics Project [36,37], and Zeller et al. [38]. Clinical significance was evaluated with ClinVar/NCBI [39].

### 2.6. RNAseq

Gene expression of CD4^+^ T lymphocytes was evaluated by RNA sequencing performed with the Illumina Hi-seq platform (San Diego, CA, USA) using a paired-end 150 bp protocol. The quality of RNAseq reads was investigated using FASTQC version 0.11.5. Reads were pseudoaligned to the human transcriptome (Ensembl version 93, GRCh38), using coding and non-coding genes. Expression was quantified using Salmon version 0.11.0 with 30 bootstrap cycles. Quantification files were imported to SLEUTH version 0.29.0 and pairwise comparisons between controls and EPF patients were performed. The Log2 fold change was obtained with the Wald test. All statistical analyses were performed using R version 3.4.4. *p* < 0.005 was established as significant [21].

## 3. Results

### 3.1. Genetic Association of Histone (De)Acetylation-Related Genes in EPF

Three intronic SNPs in different genes were associated with EPF. One of these genes encodes an HDAC (*HDAC4*) and the other two, HDAC complex members—*GSE1* (Gse1 coiled-coil protein) and *PHF21A* (PHD zinc-finger protein 21A). Carriers of the HDAC4 rs4852054*A intronic allele (OR = 1.79 [95%CI = 1.21–2.67], *p* = 0.004) presented increased susceptibility to EPF. The GSE1 rs13339618*A regulatory allele (OR = 0.57 [95%CI = 0.41–0.80], *p* = 0.001) and the *PHF21A rs4756055 A/A* intronic genotype (OR = 0.391 [95%CI = 0.228–0.672], *p* = 0.0006) were associated with decreased EPF susceptibility. These associations were not replicated in SPF samples, suggesting different etiologies for EPF and SPF forms (Table 1). This may indicate distinctions in the pathogenesis of EPF and SPF.

### 3.2. Predicted Consequences of SNPs in Histone (De)Acetylation-Related Genes Associated with EPF

Concerning functional annotation, HDAC4_rs4852054*A and GSE1_rs13339618*A present regulatory features, being located on a promoter flanking region and an enhancer, respectively [23]. 

*HDAC4* presents two similar transcripts (differing by five codons), expressed at similar levels in most tissues. In the blood, *HDAC4* is mostly expressed in granulocytes (neutrophils—47.0 normalized transcript per million, nTPM, and basophils—32.0 nTPM on the HPA dataset, 26.0 nTPM in the Monaco dataset), and much less so in the myeloid and lymphocytic lineages (very low in HPA, between 5 and 12 nTPM in monocytes and 1 and 10 nTPM in different kinds of T cells of the Monaco dataset) [24,25]. Its expression in the skin is rather low, at 11.1 nTPM in 1734 suprabasal keratinocyte cells [24]. Even so, the region containing HDAC4_rs4852054*A is enriched for enhancer- (H3K4me1 and H3K27ac) and promoter-associated histone marks (H3K9ac) and/or open-chromatin (H3K36, DNAse) in immune (common myeloid progenitor CD34^+^ cells, monocytes, and T cells) and skin cells (fibroblasts, normal human keratinocytes, and melanocyte primary cells) (Table S3, with data from Haploreg v.4.1, Roadmap and ENCODE [27,29,30]). The region with this allelic variant is occupied by the NFIC regulatory protein in the myeloid K562 cell lineage [30]. Furthermore, HDAC4_rs4852054*A is in high LD (r^2^ > 0.90) with six other *HDAC4* polymorphisms [31]. An *HDAC4* haplotype (composed of six SNPs in almost absolute LD) is associated with higher *HDAC4-AS1* (HDAC4 antisense RNA 1) gene expression in skin and/or blood cells [35] (Table 2). 

*GSE1* encodes five transcripts in the skin, whose expression is not significantly affected by sun exposure [35]. In the skin, *GSE1* is expressed in suprabasal keratinocytes (29.1 nTPM in 1734 cells), B cells (28.8 nTPM in 268 skin B cells), and macrophages (31.2 nTPM in 961 skin macrophages, 24.5 nTPM in 1776 Langerhans cells). In lymph nodes, its expression in two B cell populations doubles (50.7–53.6 nTPM, 2283–2025 B cells) [24]. The region containing GSE1_rs13339618*A is enriched with enhancer- (H3K4me1 and H3K27ac) and/or promoter-associated histone marks (H3K4m3) in skin cells (fibroblasts and melanocyte primary cells) (Table S3). It is also occupied by the ZBTB33 and REST regulatory proteins in the K562 myeloid cell lineage [30]. GSE1_rs13339618*A presents high LD with seven other *GSE1* alleles (Table 2) [31]. These alleles, except GSE1_rs8060638*A and GSE1_rs28671512*T, are associated with five times higher *GSE1* gene expression in blood cells [33]. GSE1_rs13329722*C and GSE1_rs7498141*A are also associated with *TRIM35* (tripartite motif containing 35) gene expression in monocytes [38] (Table 2).

*PH21A* presents a moderate expression of five transcript variants in the skin, regardless of sun exposure. Skin-resident Langerhans cells, T and B cells, as well as macrophages, all present similar expression of this gene (varying from 12.6 to 16.5 nTPM in Langerhans cells and T cells, respectively). Its expression is relatively higher in lymph node B cells (27.4–48.1 nTPM) and T cells (20.0–34.2 nTPM) [24]. The PHF21A_rs4756055*A region is enriched with enhancers (H3K4me1 and H3K27ac), promoters (H3K4me3 and H3K9ac), and/or open-chromatin (DNase) in immune (neutrophils, monocytes, and mononuclear, natural killer, T and B cells) and skin cells (fibroblasts, keratinocytes, and melanocytes) [27,29] (Table S3). This region is also occupied by myocyte enhancer factor 2B (MEF2B), paired box 8 (PAX8), and nuclear factor of activated T cells 3 (NFATC3) regulatory proteins in the GM12878 cell line [30]. PHF21A_rs4756055*A is in high LD with seven other *PHF21* alleles (Table 2) [31]. These SNPs, excepting PHF21A_rs11374563*T, are associated with slightly higher *HNRNPH1* (heterogeneous nuclear ribonucleoprotein H1) gene expression in macrophages [37]. Three SNPs in strong LD, including the one associated with lower susceptibility to EPF, are also associated with three times higher *CRY2* (cryptochrome circadian regulator 2) expression in blood cells [33]. Three other SNPs displaying a somewhat lower LD (0.8 < r^2^ < 0.9) are associated with slightly lower *CRY2* expression in monocytes [36]. PHF21A_rs923530*T, PHF21A_rs7107550*C, and PHF21A_rs74366855*A are associated with lower *SLC37A1* (solute carrier family 37 member 1) gene expression in macrophages [37]. PHF21A_rs4756055*A is also associated with lower *PEX16* (peroxisomal biogenesis factor 16) gene expression [33] and altered *PHF21A* gene expression [34] in blood cells (Table 2). 

To date, no clinical significance has been reported for these variants [39].

### 3.3. Differential RNA Expression of Histone (De)Acetylation-Related Genes in EPF

Of the 144 genes encoding HATs and HDACs, and members of histone (de)acetylation complexes, 139 were analyzed by RNAseq. Five genes were found to be differently expressed in CD4^+^ T cells from patients with EPF without oral corticoid treatment and presenting active disease, compared to controls from PF endemic regions: *ZEB2* (zinc finger E-box binding homeobox 2), *KAT2B* (lysine acetyltransferase 2B), and *PHF20* (PHD finger protein 20) were overexpressed in patients, while *KAT14* (lysine acetyltransferase 14) and *JADE1* (jade family PHD finger 1) were underexpressed (Table 3). Interestingly, *ZEB2* expression is elevated in T cells from bone marrow (229.6 nTPM), as well as from peripheral blood (248.6 nTPM). In turn, *KAT2B* is highly expressed in memory CD4^+^ T helper (Th) 1/Th17 cells, in contrast to *PHF20* (303.5 vs. 47 TPM, respectively, in the Schmiedel dataset [26]). Both may reach 80–100 nTPM in T cells of vascular tissue. *JADE1* also is highly expressed in memory CD4^+^ Th1/Th17 cells (100.2 nTPM in the Monaco dataset [25], although its expression in memory CD4^+^ T cells in the HPA dataset was lower—18.4 nTPM). *KAT14*, however, is less expressed in T cells (in these cells, its higher expression occurs in naïve CD4^+^ cells—37.3 nTPM) [24]. 

**Figure 2:**
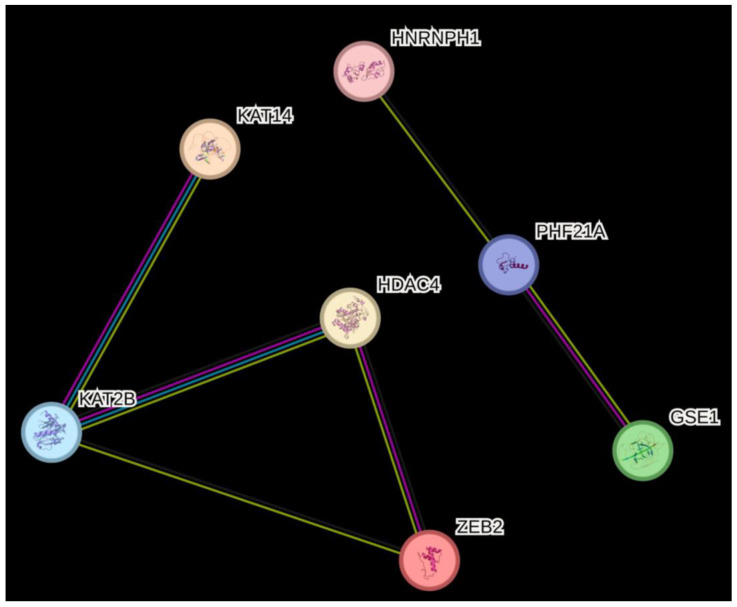
Interactions among proteins encoded by genes whose polymorphisms were associated with EPF susceptibility (*HDAC4*, *GSE1*, and *PHF21A*), have an eQTL effect (*HNRNPH1*), or which were differently expressed in EPF patients’ CD4+ T cells (*KAT14*, *ZEB2*, *KAT2B*). The interactions map was drawn using STRING (stringdb.org; accessed on 28 November 2023). The edges present a protein–protein association. Blue and purple edges indicate interactions with known co-occurrence and experimental evidence, respectively. Black and yellow edges indicate co-expression and textmining evidence, respectively. All the 3D protein structures are known or predicted.

None of them coincided with genes presenting EPF-associated variants. This is not surprising, since the associated polymorphisms do not have an eQTL effect in lymphocytes. However, HDAC4 and KAT2B are known to co-occur (may physically interact). Furthermore, HDAC4, KAT2B, and ZEB2 are known to be coexpressed, as well as HNRNPH1, PHF21A, and GSE1 (Figure 2).

## 4. Discussion

**Figure 3:**
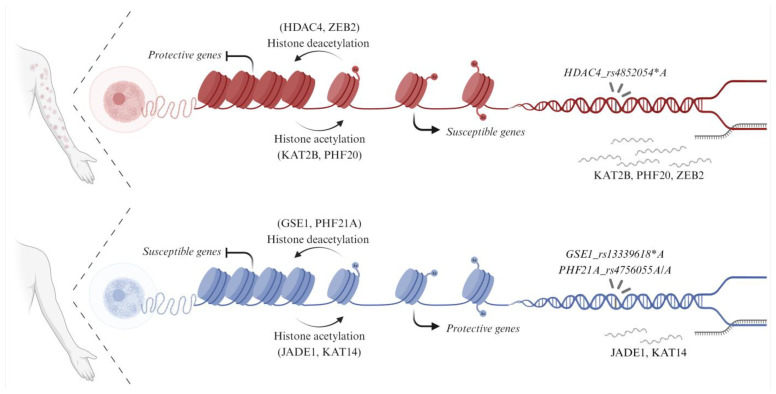
HDAC4_rs4852054*A was associated with increased EPF susceptibility, whereas GSE1_rs13339618*A and *PHF21A_ rs4756055 A/A* were associated with decreased EPF susceptibility. In CD4^+^ T cells from EPF patients, ZEB2, KAT2B, and PHF20 were overexpressed, and instead, KAT14 and JADE1 were underexpressed. By turning chromatin into a close state, deacetylation-related proteins whose polymorphisms were associated with increased EPF susceptibility (HDAC4) or which were overexpressed in patients’ CD4^+^ T cells (ZEB2) are thought to repress the expression of genes conferring protection to EPF in patients, whereas those associated with decreased EPF susceptibility (GSE1 and PHF21A) are thought to repress the expression of genes conferring susceptibility to the disease in controls. Conversely, in turning chromatin into an open state, acetylation-related proteins encoded by genes overexpressed in patients’ CD4^+^ T cells (KAT2B and PHF20) are supposed to induce expression of genes conferring susceptibility to the disease in EPF patients, while those underexpressed (JADE1 and KAT14) are supposed to induce expression of genes conferring protection to EPF in controls.

Dysregulated homeostasis in autoimmune diseases is closely associated with epigenetic modifications [13]. Altered DNA methylation and histone PTMs are known to break immune tolerance [40], but their role is still poorly understood in PF. In this work, we identified HATs, HDACs, and interacting proteins either genetically associated with EPF or differentially expressed in CD4^+^ T cells (Figure 3). Three variants within three genes related to histone deacetylase were associated with altered EPF susceptibility. The lack of association in SPF may be related to different pathophysiological pathways (reviewed in [41]).

One of the herein associated genes encodes HDAC4, a member of the human class II histone deacetylases, known to silence the transcription of genes related to the immune response [42,43,44]. It represses myocyte enhancer factors 2 A (MEF2A), C (MEF2C), and D (MEF2D) [43,44,45]. Moreover, the caspase-cleaved amino-terminal fragment of HDAC4 (independently from the HDAC4 deacetylase domain) has been shown to repress MEF2C, triggering cell death [46]. It modulates thymocyte apoptosis and T and B cell differentiation and trafficking, by regulating the expression of the steroid receptor *Nur77* [45,47] and *KLF2* (Krüppel-like factor 2) [48,49]. These HDAC4 interactions indicate a possible role for cell death in EPF physiopathology, reinforced by our results and by others [50,51]. HDAC4 is also associated with B-cell lymphoma 6 (BCL6), a repressor protein essential for germinal center B cells and T follicular helper cells (Tfh), known to be increased in the blood of bullous pemphigoid patients [52,53,54]. By the way, DNA binding sites for MEF2A and BCL6 were significantly enriched in systemic lupus erythematosus susceptibility loci [55]. HDAC4 is also associated with regulatory factor X-associated ankyrin-containing protein and class II MHC transactivator (CIITA) protein, and represses CIITA-mediated MHC class II gene expression [56]. Moreover, CIITA_rs3087456*G carriers present an increased susceptibility to EPF [57]. This variant is also an eQTL for lower CIITA expression in sun-exposed skin [35]. Finally, HDAC4 forms a complex with GATA binding protein 3 and yin-yang 1 protein and downregulates *IL5* (interleukin 5) expression [58], thus influencing Th2 cell differentiation and function. In the initial phase of the EPF immune response, Th1 and Th2 cytokines have been proposed to drive IgG1 and IgG4 anti-DSG1 production, while during the second phase, Th2 cytokines seem to drive IgG4 anti-DSG1 production [59,60].

HDAC4_rs4852054*A carriers were approximately 1.8 times more susceptible to EPF. This variant occurs within a five SNP haplotype consistently associated with higher expression of the *HDAC4-AS1* lncRNA in blood and skin cells [35]. On hypoxia, this antisense inhibits *HDAC4* expression in the human retinal pigment epithelial ARPE-19 cell line [61]. If this regulation also occurs in immune cells, higher *HDAC4-AS1* expression in these individuals may ultimately decrease *HDAC4* expression and activity, maintaining aberrant gene activation in the autoimmune response.

The other two polymorphisms associated with decreased EPF susceptibility are also associated with altered expression of histone deacetylase complex members, namely of two BRAF35-HDAC chromatin-remodeling complex components in blood: GSE1 and PHF21A [62,63]. GSE1_rs13339618*A is associated with greatly enhanced expression of *GSE1* in blood cells, probably of the myeloid lineage. This higher gene expression may increase the efficiency of acetyl group removal from histones in inflammatory genes, reducing the odds of autoimmunity. GSE1_rs13339618*A is also associated with altered *TRIM35* expression [38]. Interestingly, TRIM35 negatively regulates type I interferon response by interacting with IRF7, promoting its ubiquitination and subsequent degradation [64]. Cells and pathways with a pathogenic role in autoimmunity can be activated by type I interferon [65].

PHF21A regulates IL-1-induced gene expression [66] and is a core protein of the CoREST complex [67], whose inhibition impairs the suppressive function of regulatory T (Treg) cells [68]. These findings support the notion that dysfunctional Treg cells and cytokine production contribute to EPF pathophysiology. Furthermore, PHF21A_rs4756055*A and seven SNPs at LD are also associated with higher expression of the circadian *CRY2* and *HNRNPH1* genes in blood cells and macrophages, respectively, and with lower expression of *PEX16* and *SLC37A1* genes in the same types of cells. Thus, this region has a regulatory influence on several genes in immune cells, which may also be implicated in EPF. *CRY2* expression is also reduced in celiac disease [69], and *Cry* double knockout mice have an overactivated B cell receptor signaling pathway, contributing to high serum IgG concentrations and antinuclear antibodies, with severe lung and kidney involvement [70]. Thus, higher *CRY2* expression in PHF21A_rs4756055*A carriers may improve resistance to autoimmunity. Moreover, HNRNPH1 has been shown to reduce *IL8* expression [71]. Interestingly, higher levels of IL-8 were found in the serum and blister fluids of patients with pemphigus [72]. The possible involvement of the other genes remains elusive.

Five genes were differentially expressed in CD4^+^ T cells from patients with active EPF without oral corticoid treatment. Three of these were overexpressed in patients—*ZEB2*, *KAT2B*, and *PHF20*, while the other two were underexpressed—*KAT14* and *JADE1.*

ZEB2, also known as Smad-interacting protein 1, is a member of two-handed zinc finger/homeodomain transcription factors [73], which binds E box sequences and interacts with several co-repressors containing HDACs [74]. ZEB2 is essential for normal hematopoietic stem cell and hematopoietic progenitor cell differentiation and mobilization [75]. It interacts with T-Box Transcription Factor 21 and represses *IL7R* and *IL2* expression, promoting terminal differentiation of lymphocytes, especially of CD8^+^ T cells [76,77]. It also regulates dendritic cell differentiation [78]. The consequences of its overexpression in CD4^+^ T cells from EPF patients warrant further investigation.

Consistent with our findings, the HDAC *KAT2B* gene is also upregulated in PBMCs from rheumatoid arthritis patients [79], and its overexpression increases histone H4 acetylation on the enhancer of MHC class I promoters [80]. It is nevertheless also important in Treg cell development [68,81,82]. KAT2B interacts with KLF10 and induces *FOXP3* transcription [68]. Indeed, decreased levels of CD4^+^ FOXP3^+^ T cells occur in *Kat2b*-deficient mice [82,83]. Moreover, KAT2B acetylates the *IL2* promoter, protecting Treg cells from undergoing apoptosis upon T cell receptor stimulation [81]. It even acetylates FOXP3 itself [81] and DEK protein [84]. When overexpressed, KAT2B acetylates DEK, reducing its affinity for DNA and impairing its transcription repression function [84]. Interestingly, anti-DEK autoantibodies have been identified in juvenile rheumatoid arthritis [85] and other inflammatory diseases [86]. Thus, KAT2B overexpression in T lymphocytes may maintain a deregulated immune response in EPF patients.

In contrast to ZEB2 and KAT2B, which are related to histone deacetylation, PHF20 is a component of the NSL (non-specific lethal) complex, which has HAT activity [87]. *Phf20* knockout impairs thymocyte differentiation and maturation in mice [88]. Moreover, the second Tudor domain and the PHD finger of PHF20 recognize dimethylated lysines on p53, promoting nuclear factor kappa B (NF-κB) transcriptional activity [88,89,90], by stabilizing p53 [91] and avoiding its acetylation by the NSL complex [90]. PHF20 further regulates autophagy genes [92]. Its higher expression possibly implicates this protein in EPF autoimmune activation.

Among the two genes with lower expression in CD4^+^ T lymphocytes of patients, KAT14 is a subunit of the ADA2A-contaning acetyltransferase complex, essential for mammalian development, exerting a role in cell cycle progression and apoptosis [93]. Its impact on EPF autoimmune activation should be further investigated.

Jade family PHD finger 1 (JADE1, also known as PHF17) belongs to a subfamily of PHD zinc finger proteins, which contain two PHD zinc finger domains. JADE1 is subjected to post-transcriptional regulation and polyadenylation, resulting in multiple transcripts and two protein isoforms: the major isoform JADE1L and the truncated isoform JADE1S [94]. JADE1 is a transcriptional co-activator associated with HAT activity specific for histone H4 [95]. It interacts with lysine acetyltransferase 5 and 7 (KAT5 and KAT7, known as TIP60 and HBO1, respectively), which are close homologs, but is only able to induce transcription with KAT7 [95,96]. Essential roles of JADE1 in cell cycle regulation [97], and also as a consequence on apoptosis [98], have been described.

## 5. Conclusions

This is the first study suggesting that disruption of histone (and other proteins) (de)acetylation is involved in EPF pathophysiology, indicating a possible epigenetic effect in the autoimmune response.

We suggest that by turning chromatin into a close state, deacetylation-related proteins associated with increased EPF susceptibility (HDAC4) or overexpressed in patients’ CD4^+^ T cells (ZEB2) repress the expression of protective genes. Conversely, those associated with decreased EPF susceptibility (GSE1 and PHF21A) may repress the expression of genes conferring susceptibility to the disease. In the opposite direction, by turning chromatin into an open state, acetylation-related proteins overexpressed in patients’ CD4^+^ T cells (KAT2B and PHF20) may induce the expression of genes conferring susceptibility to the disease. Furthermore, those underexpressed (JADE1 and KAT14) induce the expression of protective genes. Some of these genes, whose expression is supposedly regulated by histone (de)acetylase-related proteins, seem to be essential genes in immune cell differentiation and cell death processes (Figure 3).

In conclusion, our findings highlight the importance of histone (de)acetylation dynamic processes, and its crosstalk with other histone PTMs, such as (de)methylation, in the autoimmune pathophysiology of EPF. Experiments evaluating the chromatin state may increase our understanding of epigenetic alterations in PF.

## Figures and Tables

**Table 1:** Genetic association analysis with endemic and sporadic pemphigus foliaceus.

Gene	SNP	Endemic Pemphigus Foliaceus								Sporadic Pemphigus Foliaceus							
		MAF (%)		Model	Control	Patients	OR	95% CI	*p*	MAF (%)		Model	Control	Patients	OR	95% CI	*p*
		Controls	Patients							Controls	Patients						
HDAC4	rs4852054	30.67	39.82	ADD	119/269	180/272	1.525	[1.14–2.05]	0.0051	18.71	23.03	ADD	52/226	35/117	1.299	[0.80–2.11]	0.2891
2q37.3	G > a			REC	19/175	32/194	1.536	[0.84–2.81]	0.1647			REC	05/134	04/72	1.489	[0.39–5.72]	0.5621
intron 1				**DOM**	**78/148**	**94/100**	**1.794**	**[1.21–2.67]**	**0.0038**			DOM	47/92	31/45	1.348	[0.76–2.40]	0.3097
GSE1	rs13339618	29.64	19.42	**ADD**	**115/273**	**87/361**	**0.571**	**[0.41–0.80]**	**0.0011**	19.06	23.65	ADD	53/225	35/133	1.257	[0.81–1.96]	0.3108
16q24.1	G > a			REC	16/178	5/219	0.261	[0.09–0.73]	0.0102			REC	09/130	07/67	1.509	[0.54–4.23]	0.4339
regulatory region downstream				DOM	99/95	82/142	0.578	[0.39–0.86]	0.0064			DOM	44/95	28/46	1.314	[0.73–2.37]	0.3643
PHF21A	rs4756055	38.05	42.82	ADD	186/198	172/280	0.671	[0.50–0.90]	0.0079	45.32	37.50	ADD	126/152	57/95	0.740	[0.50–1.10]	0.1322
11p11.2	g > A			**REC**	**50/142**	**26/200**	**0.391**	**[0.23–0.67]**	**0.0007**			REC	31/108	12/64	0.653	[0.38–1.36]	0.2559
intron 1				DOM	136/56	146/80	0.792	[0.52–1.21]	0.2831			DOM	95/44	45/31	0.672	[0.38–1.20]	0.1801

In bold, significant associations (*p* < 0.005). The minor alleles in our sample are given in lowercase; they are the reference for the associations. Abbreviations: ADD, additive model; CI, confidence interval; DOM, dominant model; GSE1, Gse1 coiled-coil protein; HDAC4, histone deacetylase 4; MAF, minor allele frequency; OR, odds ratio; PHF21A, PHD finger protein 21A; REC, recessive model; SNP, single nucleotide polymorphism.

**Table 2:** Alleles in high LD with *HDAC4*, *GSE1*, and *PHF21A* alleles associated with EPF and their eQTL effect in relevant tissues and cells for PF pathophysiology.

SNPs	r^2^	eQTL	Tissues and Cells
HDAC4_rs4852054*A (Susceptibility)	-	*HDAC4-AS1*	Blood (ES = 0.15; *p* = 1.5 × 10^−4^)
			Skin not sun-exposed (ES = 0.17; *p* = 3.4 × 10^−3^)
HDAC4_rs4852053*C	1.0	*HDAC4-AS1*	Blood (ES = 0.17, *p* = 4.7 × 10^−5^)
			Skin sun-exposed (ES = 0.16; *p* = 5.0 × 10^−3^)
HDAC4_rs55932933*T	0.99	*HDAC4-AS1*	Blood (ES = 0.17; *p* = 6.3 × 10^−5^)
			Skin sun-exposed (ES = 0.16; *p* = 5.0 × 10^−3^)
HDAC4_rs56064197*T	0.99	*HDAC4-AS1*	Blood (ES = 0.17; *p* = 5.8 × 10^−5^)
			Skin sun-exposed (ES = 0.16; *p* = 5.0 × 10^−3^)
HDCA4_rs1476321*T	0.99	*HDAC4-AS1*	Blood (ES = 0.15; *p* = 1.9 × 10^−4^)
			Skin not sun-exposed (ES = 0.17; *p* = 3.4 × 10^−3^)
HDCA4_rs10182344*A	0.99	*HDAC4-AS1*	Blood (ES = 0.14; *p* = 3.5 × 10^−4^)
HDCA4_rs58332998*A	0.93	-	-
GSE1_rs13339618*A (Protection)		*GSE1*	Blood (ES = 5.37; *p* = 7.9 × 10^−8^)
GSE1_rs13339626*A	1.0	*GSE1*	Blood (ES = 5.37; *p* = 7.9 × 10^−8^)
GSE1_rs13329722*C	1.0	*GSE1*	Blood (ES = 5.40; *p* = 6.9 × 10^−8^)
		*TRIM35*	Monocytes (EF = -; *p* = 9.2 × 10^−6^)
GSE1_rs7498141*A	1.0	*GSE1*	Blood (EF = 5.40; *p* = 6.9 × 10^−8^)
		*TRIM35*	Monocytes (ES = -; *p* = 9.0 × 10^−6^)
GSE1_rs4843505*G	1.0	*GSE1*	Blood (ES = 5.40; *p* = 6.9 × 10^−8^)
GSE1_rs13332576*A	0.99	*GSE1*	Blood (ES = 5.31; *p* = 1.1 × 10^−7^)
GSE1_rs8060638*A	0.89	-	-
GSE1_rs28671512*T	0.99	-	-
PHF21A_rs4756055*A/A (Protection)	-	*CRY2*	Blood (ES = 3.64; *p* = 2.7 × 10^−4^)
		*PEX16*	Blood (ES = −3.16; *p* = 1.0 × 10^−3^)
		*PHF21A*	Blood (ES = -; *p* = 2.8 × 10^−4^)
		*HNRNPH1*	Macrophages (ES = 0.08; *p* = 5.8 × 10^−6^)
PHF21A_rs1976182*A	0.98	*HNRNPH1*	Macrophages (ES = 0.08; *p* = 5.4 × 10^−6^)
PHF21A_rs923530*T	0.87	*CRY2*	Monocytes (ES = −0.04, *p* = 3.8 × 10^−4^)
		*HNRNPH1*	Macrophages (ES = 0.08, *p* = 7.6 × 10^−6^)
		*SLC37A1*	Macrophages (EF = −0.05, *p* = 2.3 × 10^−6^)
PHF21A_rs11374563*T	0.87	-	-
PHF21A_rs7109480*T	0.93	*CRY2*	Blood (ES = 3.46, *p* = 5.4 × 10^−4^)
		*HNRNPH1*	Macrophages (EF = 0.08; *p* = 6.8 × 10^−6^)
PHF21A_rs7107550*C	0.86	*CRY2*	Monocytes (ES = −0.04, *p* = 3.2 × 10^−4^)
		*HNRNPH1*	Macrophages (ES = 0.08, *p* = 9.3 × 10^−6^)
		*SLC37A1*	Macrophages (EF = −0.05, *p* = 4.3 × 10^−6^)
PHF21A_rs950105*A	1.0	*CRY2*	Blood (ES = 3.41; *p* = 6.4 × 10^−4^)
		*HNRNPH1*	Macrophages (EF = 0.08; *p* = 9.1 × 10^−6^)
PHF21A_rs74366855*A	0.87	*CRY2*	Monocytes (ES = −0.04, *p* = 5.8 × 10^−4^)
		*HNRNPH1*	Macrophages (ES = 0.08, *p* = 8.0 × 10^−6)^
		*SLC37A1*	Macrophages (EF = −0.05, *p* = 5.7 × 10^−6^)

High LD (r^2^ > 0.80) between SNPs in the European population was evaluated with LDLink [31]. eQTL effect (*p* ≤ 5.0 × 10^−3^) was observed in Genehopper Qtlizer [32], which compiles information from Blood eQTL [33], GRASP 2 Catalog [34], GTEx v8 [35], The Cardiogenics Project [36,37], and Zeller et al. [38]. “-” indicates missing/unfound data. Abbreviations: CRY2, cryptochrome circadian regulator 2; ES, effect size; EPF, endemic pemphigus foliaceus; eQTL, expression quantitative trait loci; GSE1, Gse1 coiled-coil protein; HDAC4, histone deacetylase 4; HDAC4-AS1, HDAC4 antisense RNA 1; HNRNPH1, heterogeneous nuclear ribonucleoprotein H1; LD, linkage disequilibrium; PEX16, peroxisomal biogenesis factor 16; PF, pemphigus foliaceus; PHF21A, PHD finger protein 21A; SLC37A1, solute carrier family 37 member 1; SNP, single nucleotide polymorphism; TRIM35, tripartite motif containing 35.

**Table 3:** (De)acetylation-related genes differently expressed in CD4^+^ T cells of endemic pemphigus foliaceus patients compared to controls.

Gene	Full Name	Fold Change	*p*-Value
*ZEB2*	zinc finger E-box binding homeobox 2	1.08	6.2 × 10^−6^
*KAT2B*	lysine acetyltransferase 2B	0.55	2.8 × 10^−5^
*PHF20*	PHD finger protein 20	0.41	8.6 × 10^−5^
*KAT14*	lysine acetyltransferase 14	−0.59	2.0 × 10^−4^
*JADE1*	jade family PHD finger 1	−0.31	2.6 × 10^−3^

PF patients presented new skin lesions coming up constantly, before starting oral corticosteroid treatment. Controls were from endemic areas for PF.

## Data Availability

The data presented in this study are available on request from the corresponding author. The data are not publicly available due to ethical restrictions.

## Supplementary Materials

The following supporting information can be downloaded at: https://www.mdpi.com/article/10.3390/life14010060/s1, Table S1: Genomic position of HATs, HDACs and histone (de)acetylation complexes members; Table S2: Primer sequences used for investigating genetic association in sporadic pemphigus foliaceus samples; Table S3: Epigenomic annotation of allelic variants associated with PF in immune and skin cells.

## References

[1] Kasperkiewicz M., Ellebrecht C.T., Takahashi H., Yamagami J., Zillikens D., Payne A.S., Amagai M. (2017). Pemphigus. Nat. Rev. Dis. Primers.

[2] Schmidt E., Kasperkiewicz M., Joly P. (2019). Pemphigus. Lancet.

[3] Qian Y., Jeong J.S., Maldonado M., Valenzuela J.G., Gomes R., Teixeira C., Evangelista F., Qaqish B., Aoki V., Hans G. (2012). Cutting Edge: Brazilian Pemphigus Foliaceus Anti-Desmoglein 1 Autoantibodies Cross-React with Sand Fly Salivary LJM11 Antigen. J. Immunol..

[4] Petzl-Erler M.L. (2020). Beyond the HLA Polymorphism: A Complex Pattern of Genetic Susceptibility to Pemphigus. Genet. Mol. Biol..

[5] Spadoni M.B., Bumiller-Bini V., Petzl-Erler M.L., Augusto D.G., Boldt A.B.W. (2020). First Glimpse of Epigenetic Effects on Pemphigus Foliaceus. J. Investig. Dermatol..

[6] Petzl-Erler M.L., Santamaria J. (1988). Are HLA Class II Genes Controlling Susceptibility and Resistance to Brazilian Pemphigus Foliaceus (Fogo Selvagem)?. Tissue Antigens.

[7] Lobo-Alves S.C., Augusto D.G., Magalhães W.C.S., Tarazona-Santos E., Lima-Costa M.F., Barreto M.L., Horta B.L., de Almeida R.C., Petzl-Erler M.L. (2019). Long Noncoding RNA Polymorphisms Influence Susceptibility to Endemic Pemphigus Foliaceus. Br. J. Dermatol..

[8] Salviano-Silva A., Farias T.D.J., Bumiller-Bini V., Castro M.D.S., Lobo-Alves S.C., Busch H., Pföhler C., Worm M., Goebeler M., van Beek N. (2021). Genetic Variability of Immune-Related LncRNAs: Polymorphisms in LINC-PINT and LY86-AS1 Are Associated with Pemphigus Foliaceus Susceptibility. Exp. Dermatol..

[9] Salviano-Silva A., Becker M., Augusto D.G., Busch H., Adelman Cipolla G., Farias T.D.J., Bumiller-Bini V., Calonga-Solís V., Munz M., Franke A. (2021). Genetic Association and Differential Expression of HLA Complex Group LncRNAs in Pemphigus. J. Autoimmun..

[10] Allis C.D., Caparros M.-L., Jenuwein T., Lachner M., Reinberg D., Allis C.D., Caparros M.-L., Jenuwein T., Reinberg D., Lachner M. (2015). Overview and Concepts. Epigenetics.

[11] Marmorstein R., Zhou M.M. (2014). Writers and Readers of Histone Acetylation: Structure, Mechanism, and Inhibition. Cold Spring Harb. Perspect. Biol..

[12] Zhao M., Wang Z., Yung S., Lu Q. (2015). Epigenetic Dynamics in Immunity and Autoimmunity. Int. J. Biochem. Cell Biol..

[13] Zhou Y., Su Y., Lu Q., Hu N., Qiu X., Luo Y., Yuan J., Li Y., Lei W., Zhang G. (2008). Abnormal Histone Modification Patterns in Lupus CD_4_+ T Cells. J. Rheumatol..

[14] Zhang Z., Song L., Maurer K., Petri M.A., Sullivan K.E. (2010). Global H_4_ Acetylation Analysis by ChIP-Chip in Systemic Lupus Erythematosus Monocytes. Genes. Immun..

[15] Zhao M., Liang G., Wu X., Wang S., Zhang P., Su Y., Yin H., Tan Y., Zhang J., Lu Q. (2012). Abnormal Epigenetic Modifications in Peripheral Blood Mononuclear Cells from Patients with Alopecia Areata. Br. J. Dermatol..

[16] Zhao M., Huang W., Zhang Q., Gao F., Wang L., Zhang G., Su Y., Xiao R., Zhang J., Tang M. (2012). Aberrant Epigenetic Modifications in Peripheral Blood Mononuclear Cells from Patients with Pemphigus Vulgaris. Br. J. Dermatol..

[17] Calonga-Solís V., Amorim L.M., Farias T.D.J., Petzl-Erler M.L., Malheiros D., Augusto D.G. (2021). Variation in Genes Implicated in B-Cell Development and Antibody Production Affects Susceptibility to Pemphigus. Immunology.

[18] Hoch V.B.B., Kohler A.F., Augusto D.G., Lobo-Alves S.C., Malheiros D., Cipolla G.A., Boldt A.B.W., Braun-Prado K., Wittig M., Franke A. (2022). Genetic Associations and Differential MRNA Expression Levels of Host Genes Suggest a Viral Trigger for Endemic Pemphigus Foliaceus. Viruses.

[19] Augusto D.G., de Almeida R.C., Farias T.D.J., Magalhães W.C.S., Malheiros D., Lima-Costa M.F., Barreto M.L., Horta B.L., Kumar V., Wittig M. (2021). Unsuspected Associations of Variants within the Genes NOTCH4 and STEAP2-AS1 Uncovered by a GWAS in Endemic Pemphigus Foliaceus. J. Investig. Dermatol..

[20] Maglott D., Ostell J., Pruitt K.D., Tatusova T. (2005). Entrez Gene: Gene-centered information at NCBI. Nucleic Acids Res..

[21] Benjamin D.J., Berger J.O., Johannesson M., Nosek B.A., Wagenmakers E.J., Berk R., Bollen K.A., Brembs B., Brown L., Camerer C. (2018). Redefine Statistical Significance. Nat. Hum. Behav..

[22] Kent W.J., Sugnet C.W., Furey T.S., Roskin K.M., Pringle T.H., Zahler A.M., Haussler A.D. (2002). The Human Genome Browser at UCSC. Genome Res..

[23] Yates A.D., Achuthan P., Akanni W., Allen J., Allen J., Alvarez-Jarreta J., Amode M.R., Armean I.M., Azov A.G., Bennett R. (2020). Ensembl 2020. Nucleic Acids Res..

[24] Uhlén M., Fagerberg L., Hallström B.M., Lindskog C., Oksvold P., Mardinoglu A., Sivertsson Å., Kampf C., Sjöstedt E., Asplund A. (2015). Tissue-Based Map of the Human Proteome. Science.

[25] Monaco G., Lee B., Xu W., Mustafah S., Hwang Y.Y., Carré C., Burdin N., Visan L., Ceccarelli M., Poidinger M. (2019). RNA-Seq Signatures Normalized by MRNA Abundance Allow Absolute Deconvolution of Human Immune Cell Types. Cell Rep..

[26] Schmiedel B.J., Singh D., Madrigal A., Valdovino-Gonzalez A.G., White B.M., Zapardiel-Gonzalo J., Ha B., Altay G., Greenbaum J.A., McVicker G. (2018). Impact of Genetic Polymorphisms on Human Immune Cell Gene Expression. Cell.

[27] Ward L.D., Kellis M. (2012). HaploReg: A Resource for Exploring Chromatin States, Conservation, and Regulatory Motif Alterations within Sets of Genetically Linked Variants. Nucleic Acids Res..

[28] Oscanoa J., Sivapalan L., Gadaleta E., Dayem Ullah A.Z., Lemoine N.R., Chelala C. (2020). SNPnexus: A Web Server for Functional Annotation of Human Genome Sequence Variation (2020 Update). Nucleic Acids Res..

[29] Kundaje A., Meuleman W., Ernst J., Bilenky M., Yen A., Heravi-Moussavi A., Kheradpour P., Zhang Z., Wang J., Roadmap Epigenomics Consortium (2015). Integrative Analysis of 111 Reference Human Epigenomes. Nature.

[30] Dunham I., Kundaje A., Aldred S.F., Collins P.J., Davis C.A., Doyle F., Epstein C.B., Frietze S., Harrow J., Kaul R. (2012). An Integrated Encyclopedia of DNA Elements in the Human Genome. Nature.

[31] Myers T.A., Chanock S.J., Machiela M.J. (2020). LDlinkR: An R Package for Rapidly Calculating Linkage Disequilibrium Statistics in Diverse Populations. Front. Genet..

[32] Munz M., Tönnies S., Balke W.T., Simon E. (2015). Multidimensional Gene Search with Genehopper. Nucleic Acids Res..

[33] Westra H.J., Peters M.J., Esko T., Yaghootkar H., Schurmann C., Kettunen J., Christiansen M.W., Fairfax B.P., Schramm K., Powell J.E. (2013). Systematic Identification of Trans EQTLs as Putative Drivers of Known Disease Associations. Nat. Genet..

[34] Fehrmann R.S.N., Jansen R.C., Veldink J.H., Westra H.J., Arends D., Bonder M.J., Fu J., Deelen P., Groen H.J.M., Smolonska A. (2011). Trans-Eqtls Reveal That Independent Genetic Variants Associated with a Complex Phenotype Converge on Intermediate Genes, with a Major Role for the Hla. PLoS Genet..

[35] Lonsdale J., Thomas J., Salvatore M., Phillips R., Lo E., Shad S., Hasz R., Walters G., Garcia F., Young N. (2013). The Genotype-Tissue Expression (GTEx) Project. Nat. Genet..

[36] Garnier S., Truong V., Brocheton J., Zeller T., Rovital M., Wild P.S., Ziegler A., Munzel T., Tiret L., Blankenberg S. (2013). Genome-Wide Haplotype Analysis of Cis Expression Quantitative Trait Loci in Monocytes. PLoS Genet..

[37] Codoni V., Blum Y., Civelek M., Proust C., Franzén O., Consortium C., Björkegren J.L.M., Le Goff W., Cambien F., Lusis A.J. (2016). Preservation Analysis of Macrophage Gene Coexpression between Human and Mouse Identifies PARK2 as a Genetically Controlled Master Regulator of Oxidative Phosphorylation in Humans. G3 Genes. Genomes Genet..

[38] Zeller T., Wild P., Szymczak S., Rotival M., Schillert A., Castagne R., Maouche S., Germain M., Lackner K., Rossmann H. (2010). Genetics and beyond—the Transcriptome of Human Monocytes and Disease Susceptibility. PLoS ONE.

[39] Landrum M.J., Chitipiralla S., Brown G.R., Chen C., Gu B., Hart J., Hoffman D., Jang W., Kaur K., Liu C. (2020). ClinVar: Improvements to Accessing Data. Nucleic Acids Res..

[40] Meda F., Folci M., Baccarelli A., Selmi C. (2011). The Epigenetics of Autoimmunity. Cell Mol. Immunol..

[41] Bumiller-Bini V., Cipolla G.A., de Almeida R.C., Petzl-Erler M.L., Augusto D.G., Boldt A.B.W. (2018). Sparking Fire under the Skin? Answers from the Association of Complement Genes with Pemphigus Foliaceus. Front. Immunol..

[42] Grozinger C.M., Hassig C.A., Schreiber S.L. (1999). Three Proteins Define a Class of Human Histone Deacetylases Related to Yeast Hda1p. Proc. Natl. Acad. Sci. USA.

[43] Miska E.A., Karlsson C., Langley E., Nielsen S.J., Pines J., Kouzarides T. (1999). HDAC4 Deacetylase Associates with and Represses the MEF2 Transcription Factor. EMBO J..

[44] Wang A.H., Bertos N.R., Vezmar M., Pelletier N., Crosato M., Heng H.H., Th’ng J., Han J., Yang X.-J. (1999). HDAC4, a Human Histone Deacetylase Related to Yeast HDA1, Is a Transcriptional Corepressor. Mol. Cell Biol..

[45] Youn H.D., Grozinger C.M., Liu J.O. (2000). Calcium Regulates Transcriptional Repression of Myocyte Enhancer Factor 2 by Histone Deacetylase 4. J. Biol. Chem..

[46] Paroni G., Mizzau M., Henderson C., Giannino, Sal D., Schneider C., Brancolini C. (2004). Caspase-Dependent Regulation of Histone Deacetylase 4 Nuclear-Cytoplasmic Shuttling Promotes Apoptosis. Mol. Biol. Cell.

[47] Liu Z.-G., Smith S.W., McLaughlin K.A., Schwartz L.M., Osborne B.A. (1994). Apoptotic Signals Delivered through the T-Cell Receptor of a T-Cell Hybrid Require the Immediate–Early Gene Nur_77_. Nature.

[48] Kumar A., Lin Z., SenBanerjee S., Jain M.K. (2005). Tumor Necrosis Factor Alpha-Mediated Reduction of KLF_2_ Is Due to Inhibition of MEF_2_ by NF-ΚB and Histone Deacetylases. Mol. Cell Biol..

[49] Jha P., Das H. (2017). KLF2 in Regulation of NF-ΚB-Mediated Immune Cell Function and Inflammation. Int. J. Mol. Sci..

[50] Bumiller-Bini V., Cipolla G.A., Spadoni M.B., Augusto D.G., Petzl-Erler M.L., Beltrame M.H., Boldt A.B.W. (2019). Condemned or Not to Die? Gene Polymorphisms Associated with Cell Death in Pemphigus Foliaceus. Front. Immunol..

[51] Hoch V.B.B., Schneider L., Pumpe A.E., Lüders E., Hundt J.E., Boldt A.B.W. (2022). Marked to Die-Cell Death Mechanisms for Keratinocyte Acantholysis in Pemphigus Diseases. Life.

[52] Hatzi K., Philip Nance J., Kroenke M.A., Bothwell M., Haddad E.K., Melnick A., Crotty S. (2015). BCL6 Orchestrates Tfh Cell Differentiation via Multiple Distinct Mechanisms. J. Exp. Med..

[53] Lemercier C., Brocard M.P., Puvion-Dutilleul F., Kao H.Y., Albagli O., Khochbin S. (2002). Class II Histone Deacetylases Are Directly Recruited by BCL6 Transcriptional Repressor. J. Biol. Chem..

[54] Li Q., Liu Z., Dang E., Jin L., He Z., Yang L., Shi X., Wang G. (2013). Follicular Helper T Cells (Tfh) and IL-21 Involvement in the Pathogenesis of Bullous Pemphigoid. PLoS ONE.

[55] Molineros J.E., Yang W., Zhou X.J., Sun C., Okada Y., Zhang H., Chua K.H., Lau Y.L., Kochi Y., Suzuki A. (2017). Confirmation of Five Novel Susceptibility Loci for Systemic Lupus Erythematosus (SLE) and Integrated Network Analysis of 82 SLE Susceptibility Loci. Hum. Mol. Genet..

[56] McKinsey T.A., Kuwahara K., Bezprozvannaya S., Olson E.N. (2006). Class II Histone Deacetylases Confer Signal Responsiveness to the Ankyrin-Repeat Proteins ANKRA_2_ and RFXANK. Mol. Biol. Cell.

[57] Piovezan B.Z., Petzl-Erler M.L. (2013). Both Qualitative and Quantitative Genetic Variation of MHC Class II Molecules May Influence Susceptibility to Autoimmune Diseases: The Case of Endemic Pemphigus Foliaceus. Hum. Immunol..

[58] Han S., Lu J., Zhang Y., Cheng C., Han L., Wang X., Li N., Liu C., Huang B. (2006). Recruitment of Histone Deacetylase 4 by Transcription Factors Represses Interleukin-5 Transcription. Biochem. J..

[59] Warren S.J.P., Arteaga L.A., Rivitti E.A., Aoki V., Hans-Filho G., Qaqish B.F., Lin M.S., Giudice G.J., Diaz L.A. (2003). The Role of Subclass Switching in the Pathogenesis of Endemic Pemphigus Foliaceus. J. Invest. Dermatol..

[60] Aoki V., Millikan R.C., Rivitti E.A., Hans-Filho G., Eaton D.P., Warren S.J.P., Li N., Hilario-Vargas J., Hoffmann R.G., Diaz L.A. (2004). Environmental Risk Factors in Endemic Pemphigus Foliaceus (Fogo Selvagem). JID Symp. Proc..

[61] Pan J., Zhao L. (2021). Long Non-Coding RNA Histone Deacetylase 4 Antisense RNA 1 (HDAC_4_-AS_1_) Inhibits HDAC_4_ Expression in Human ARPE-19 Cells with Hypoxic Stress. Bioengineered.

[62] Hakimi M.A., Dong Y., Lane W.S., Speicher D.W., Shiekhattar R. (2003). A Candidate X-Linked Mental Retardation Gene Is a Component of a New Family of Histone Deacetylase-Containing Complexes. J. Biol. Chem..

[63] Hakimi M.-A., Bochar D.A., Chenoweth J., Lane W.S., Mandel G., Shiekhattar R., Rosenfeld M.G. (2002). A Core-BRAF_35_ Complex Containing Histone Deacetylase Mediates Repression of Neuronal-Specific Genes. PNAS.

[64] Wang Y., Yan S., Yang B., Wang Y., Zhou H., Lian Q., Sun B. (2015). TRIM35 Negatively Regulates TLR_7_- and TLR_9_-Mediated Type I Interferon Production by Targeting IRF7. FEBS Lett..

[65] Hall J.C., Rosen A. (2010). Type I Interferons: Crucial Participants in Disease Amplification in Autoimmunity. Nat. Rev. Rheumatol..

[66] Meier-Soelch J., Jurida L., Weber A., Newel D., Kim J., Braun T., Lienhard Schmitz M., Kracht M. (2018). RNAi-Based Identification of Gene-Specific Nuclear Cofactor Networks Regulating Interleukin-1 Target Genes. Front. Immunol..

[67] Meier K., Brehm A. (2014). Chromatin Regulation: How Complex Does It Get?. Epigenetics.

[68] Xiong Y., Wang L., Giorgio E.D., Akimova T., Beier U.H., Han R., Trevisanut M., Kalin J.H., Cole P.A., Hancock W.W. (2020). Inhibiting the Coregulator CoREST Impairs Foxp3+ Treg Function and Promotes Antitumor Immunity. J. Clin. Investig..

[69] Weintraub Y., Cohen S., Yerushalmy-Feler A., Chapnik N., Tsameret S., Anafy A., Damari E., Ben-Tov A., Shamir R., Froy O. (2023). Circadian Clock Gene Disruption in White Blood Cells of Patients with Celiac Disease. Biochimie.

[70] Cao Q., Zhao X., Bai J., Gery S., Sun H., Lin D.C., Chen Q., Chen Z., Mack L., Yang H. (2017). Circadian Clock Cryptochrome Proteins Regulate Autoimmunity. Proc. Natl. Acad. Sci. USA.

[71] Tanu T., Taniue K., Imamura K., Onoguchi-Mizutani R., Han H., Jensen T.H., Akimitsu N. (2021). HnRNPH_1_-MTR_4_ Complex-Mediated Regulation of NEAT_1v2_ Stability Is Critical for IL8 Expression. RNA Biol..

[72] Kowalski E.H., Kneibner D., Kridin K., Amber K.T. (2019). Serum and Blister Fluid Levels of Cytokines and Chemokines in Pemphigus and Bullous Pemphigoid. Autoimmun. Rev..

[73] Verschueren K., Remacle J.E., Collart C., Kraft H., Baker B.S., Tylzanowski P., Nelles L., Wuytens G., Su M.T., Bodmer R. (1999). SIP1, a Novel Zinc Finger/Homeodomain Repressor, Interacts with Smad Proteins and Binds to 5′-CACCT Sequences in Candidate Target Genes. J. Biol. Chem..

[74] Postigo A.A., Dean D.C., Kipnis D.M. (2000). Differential Expression and Function of Members of the Zfh-1 Family of Zinc Fingerhomeodomain Repressors. Proc. Natl. Acad. Sci. USA.

[75] Goossens S., Janzen V., Bartunkova S., Yokomizo T., Drogat B., Crisan M., Haigh K., Seuntjens E., Umans L., Riedt T. (2011). The EMT Regulator Zeb_2_/Sip_1_ Is Essential for Murine Embryonic Hematopoietic Stem/Progenitor Cell Differentiation and Mobilization. Blood J. Am. Soc. Hematol..

[76] Omilusik K.D., Adam Best J., Yu B., Goossens S., Weidemann A., Nguyen J.V., Seuntjens E., Stryjewska A., Zweier C., Roychoudhuri R. (2015). Transcriptional Repressor ZEB_2_ Promotes Terminal Differentiation of CD_8_+ Effector and Memory T Cell Populations during Infection. J. Exp. Med..

[77] Dominguez C.X., Amezquita R.A., Guan T., Marshall H.D., Joshi N.S., Kleinstein S.H., Kaech S.M. (2015). The Transcription Factors ZEB_2_ and T-Bet Cooperate to Program Cytotoxic T Cell Terminal Differentiation in Response to LCMV Viral Infection. J. Exp. Med..

[78] Scott C.L., Soen B., Martens L., Skrypek N., Saelens W., Taminau J., Blancke G., Van Isterdael G., Huylebroeck D., Haigh J. (2016). The Transcription Factor Zeb_2_ Regulates Development of Conventional and Plasmacytoid DCs by Repressing Id_2_. J. Exp. Med..

[79] Ghasemi A., Farazmand A., Hassanzadeh V., Poursani S., Soltani S., Akhtari M., Akhlaghi M., Farhadi E., Jamshidi A., Mahmoudi M. (2023). Upregulation of KAT_2_B and ESCO_2_ Gene Expression Level in Patients with Rheumatoid Arthritis. Clin. Rheumatol..

[80] Brockmann D., Lehmkühler O., Schmücker U., Esche H. (2001). The Histone Acetyltransferase Activity of PCAF Cooperates with the Brahma/SWI_2_-Related Protein BRG-1 in the Activation of the Enhancer A of the MHC Class I Promoter. Gene.

[81] Liu Y., Bao C., Wang L., Han R., Beier U.H., Akimova T., Cole P.A., Dent S.Y.R., Hancock W.W. (2019). Complementary Roles of Gcn_5_ and Pcaf in Foxp3+ T-Regulatory Cells. Cancers.

[82] de Jong A., de Jong R.C.M., Peters E.A., Arens R., Jukema J.W., de Vries M.R., Quax P.H.A. (2021). P300/CBP Associated Factor (PCAF) Deficiency Enhances Diet-Induced Atherosclerosis in ApoE_3_*Leiden Mice via Systemic Inhibition of Regulatory T Cells. Front. Cardiovasc. Med..

[83] Bastiaansen A.J.N.M., Ewing M.M., De Boer H.C., Van Der Pouw Kraan T.C., De Vries M.R., Peters E.A.B., Welten S.M.J., Arens R., Moore S.M., Faber J.E. (2013). Lysine Acetyltransferase PCAF Is a Key Regulator of Arteriogenesis. Arter. Thromb. Vasc. Biol..

[84] Cleary J., Sitwala K.V., Khodadoust M.S., Kwok R.P.S., Mor-Vaknin N., Cebrat M., Cole P.A., Markovitz D.M. (2005). P300/CBP-Associated Factor Drives DEK into Interchromatin Granule Clusters. J. Biol. Chem..

[85] Sierakowska H., Williams K.R., Szer I.S., Szer W. (1993). The Putative Oncoprotein DEK, Part Ofa Chimera Protein Associated with Acute Myeloid Leukaemia, Is an Autoantigen in Juvenile Rheumatoid Arthritis. Clin. Exp. Immunol..

[86] Dong X., Wang J., Kabir F.N., Shaw M., Reed A.M., Stein L., Andrade L.E.C., Trevisani V.F.M., Miller M.L., Fujii T. (2000). Autoantibodies to Dek Oncoprotein in Human Inflammatory Disease. Arthritis Rheum. Off. J. Am. Coll. Rheumatol..

[87] Cai Y., Jin J., Swanson S.K., Cole M.D., Choi S.H., Florens L., Washburn M.P., Conaway J.W., Conaway R.C. (2010). Subunit Composition and Substrate Specificity of a MOF-Containing Histone Acetyltransferase Distinct from the Male-Specific Lethal (MSL) Complex. J. Biol. Chem..

[88] Badeaux A.I., Yang Y., Cardenas K., Vemulapalli V., Chen K., Kusewitt D., Richie E., Li W., Bedford M.T. (2012). Loss of the Methyl Lysine Effector Protein PHF_20_ Impacts the Expression of Genes Regulated by the Lysine Acetyltransferase MOF. J. Biol. Chem..

[89] Adams-Cioaba M.A., Li Z., Tempel W., Guo Y., Bian C., Li Y., Lam R., Min J. (2012). Crystal Structures of the Tudor Domains of Human PHF_2_0 Reveal Novel Structural Variations on the Royal Family of Proteins. FEBS Lett..

[90] Klein B.J., Wang X., Cui G., Yuan C., Botuyan M.V., Lin K., Lu Y., Wang X., Zhao Y., Bruns C.J. (2016). PHF_20_ Readers Link Methylation of Histone H_3_K_4_ and P_53_ with H_4_K_16_ Acetylation. Cell Rep..

[91] Cui G., Park S., Badeaux A.I., Kim D., Lee J., Thompson J.R., Yan F., Kaneko S., Yuan Z., Botuyan M.V. (2012). PHF_20_ Is an Effector Protein of P_53_ Double Lysine Methylation That Stabilizes and Activates P_53_. Nat. Struct. Mol. Biol..

[92] Park S.W., Kim J., Oh S., Lee J., Cha J., Lee H.S., Kim K.I., Park D., Baek S.H. (2022). PHF_20_ Is Crucial for Epigenetic Control of Starvation-Induced Autophagy through Enhancer Activation. Nucleic Acids Res..

[93] Guelman S., Kozuka K., Mao Y., Pham V., Solloway M.J., Wang J., Wu J., Lill J.R., Zha J. (2009). The Double-Histone-Acetyltransferase Complex ATAC Is Essential for Mammalian Development. Mol. Cell Biol..

[94] Tzouanacou E., Tweedie S., Wilson V. (2003). Identification of Jade1, a Gene Encoding a PHD Zinc Finger Protein, in a Gene Trap Mutagenesis Screen for Genes Involved in Anteroposterior Axis Development. Mol. Cell Biol..

[95] Panchenko M.V., Zhou M.I., Cohen H.T. (2004). Von Hippel-Lindau Partner Jade-1 Is a Transcriptional Co-Activator Associated with Histone Acetyltransferase Activity. J. Biol. Chem..

[96] Foy R.L., Ihn Y.S., Chitalia V.C., Cohen H.T., Saksouk N., Cayrou C., Vaziri C., Côté J., Panchenko M.V. (2008). Role of Jade-1 in the Histone Acetyltransferase (HAT) HBO1 Complex. J. Biol. Chem..

[97] Avvakumov N., Lalonde M.-E., Saksouk N., Paquet E., Glass K.C., Landry A.-J., Doyon Y., Cayrou C., Robitaille G.A., Richard D.E. (2012). Conserved Molecular Interactions within the HBO_1_ Acetyltransferase Complexes Regulate Cell Proliferation. Mol. Cell Biol..

[98] Zhou M.I., Foy R.L., Chitalia V.C., Zhao J., Panchenko M.V., Wang H., Cohen H.T. (2005). Jade-1, a Candidate Renal Tumor Suppressor That Promotes Apoptosis. Proc. Natl. Acad. Sci. USA.

